# Dataset and figures on time-series analysis of child restraint policy impact in Chile

**DOI:** 10.1016/j.dib.2018.11.079

**Published:** 2018-11-22

**Authors:** José Ignacio Nazif-Muñoz, Arijit Nandi, Mónica Ruiz-Casares

**Affiliations:** a Institute for Health and Social Policy and Department of Epidemiology, Biostatistics, and Occupational Health, McGill University, Montreal, Quebec, Canada; b Department of Psychiatry, McGill University, Montreal, Quebec, Canada

## Abstract

The main objective of this data article is to present the data set which depicts the impact of child restraint legislation in Chile and its regions. The population of the study consisted of all car crashes records provided by the national police from 2002 to 2014, which included children aged 0–3. Auto Regressive Integrated Moving Average ARIMA and Poisson model were used to present the association between the dependent and independent variables of interest. When the data are analyzed, it will help to determine the degree of relationship and the strength of significance between child restraint legislation policies enacted in 2005 and 2007, and child occupant fatalities and injuries. The data are related to “Impact of child restraint policies on child occupant fatalities and injuries in Chile and its regions: An interrupted time-series study” (Nazif-Munoz et al., 2018).

**Specifications table**

**Table:** 

Subject area	*Road safety*
More specific subject area	*Child restraint legislation policies*
Type of data	*Table and figures*
How data were acquired	*Police officers collect and process all traffic incidents in Chile*
Data format	*Analyzed*
Experimental factors	*Extensive national database of traffic fatalities, injuries, and crashes in Chile*
Experimental features	*The impact of child restraint legislation on child injuries and fatalities*
Data source location	*Chile*
Data accessibility	*Data come from the National Commission of Road Safety https://www.conaset.cl/programa/observatorio-datos-estadistica/*
Related research article	Nazif-Munoz, J.I., Nandi, A., Ruiz-Casares, M., 2018. Impact of child restraint policies on child occupant fatalities and injuries in Chile and its regions: An interrupted time-series study. *Accident Analysis and Prevention*, 120 pp. 38–45 [1].

**Value of the data**

• The data can be used as a platform for further investigation by other researchers interested in time series analyses.
• These data provided here can be adopted when modeling the short and long-term impact of road safety policies, including linear and non-linear forms, like in Chile [1].
• These data can be used to explore how to determine the best models when applying Auto Regressive Integrated Moving Average (ARIMA) models when assessing public policies.

## 1 Data

**Fig. 1:**
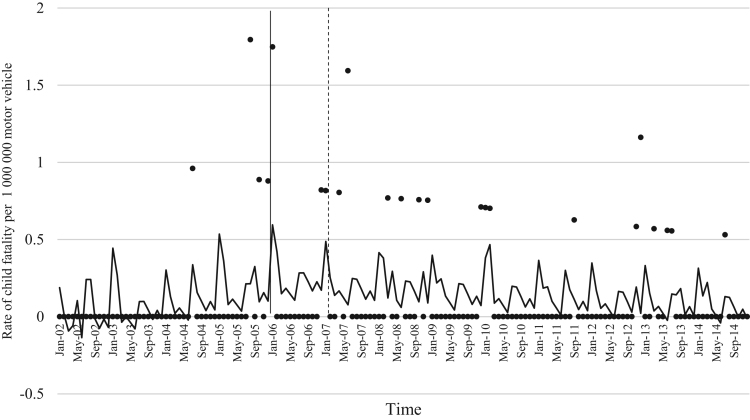
Rate of child fatalities per 1,000,000 motor vehicle in the Metropolitan Region 2002–2014. Data points represent monthly rates of child occupant fatalities aged 0–3 between 2002 and 2014. The curved lines represent fitted values for seasonally adjusted models. The full vertical line represents the month of Chile׳s CRL implementation and the broken vertical line the introduction of Chile׳s National Decree.

**Fig. 2:**
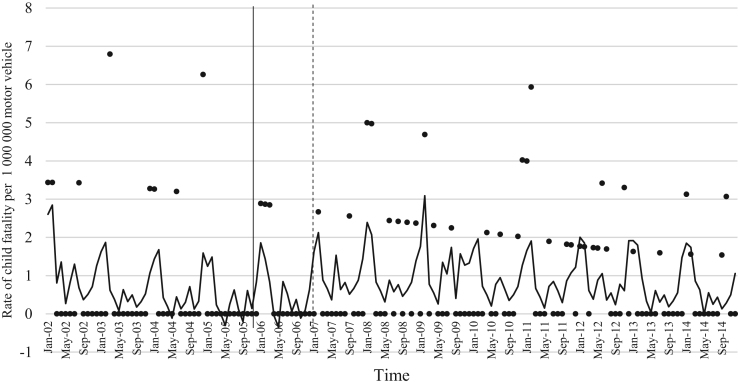
Rate of child fatalities per 1,000,000 motor vehicle in the northern regions 2002–2014. Data points represent monthly rates of child occupant fatalities aged 0–3 between 2002 and 2014. The curved lines represent fitted values for seasonally adjusted models. The full vertical line represents the month of Chile׳s CRL implementation and the broken vertical line the introduction of Chile׳s National Decree.

**Fig. 3:**
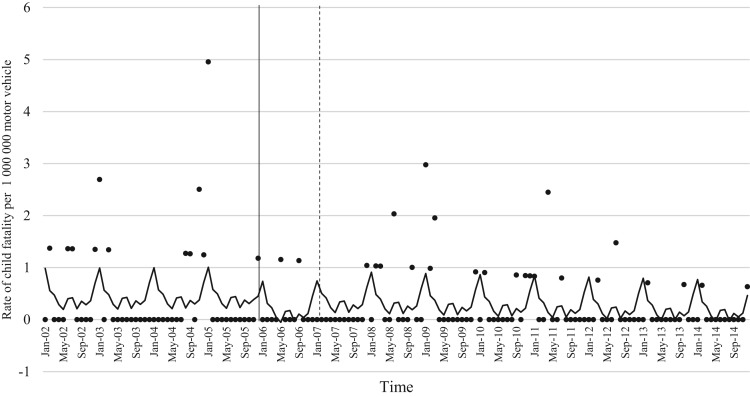
Rate of child fatalities per 1,000,000 motor vehicle in central regions 2002–2014. Data points represent monthly rates of child occupant fatalities aged 0–3 between 2002 and 2014. The curved lines represent fitted values for seasonally adjusted models. The full vertical line represents the month of Chile׳s CRL implementation and the broken vertical line the introduction of Chile׳s National Decree.

**Fig. 4:**
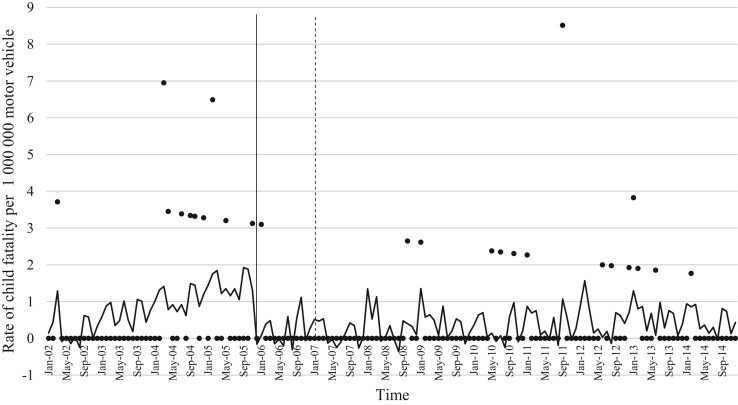
Rate of child fatalities per 1,000,000 motor vehicle in southern regions 2002–2014. Data points represent monthly rates of child occupant fatalities aged 0–3 between 2002 and 2014. The curved lines represent fitted values for seasonally adjusted models. The full vertical line represents the month of Chile׳s CRL implementation and the broken vertical line the introduction of Chile׳s National Decree.

**Fig. 5:**
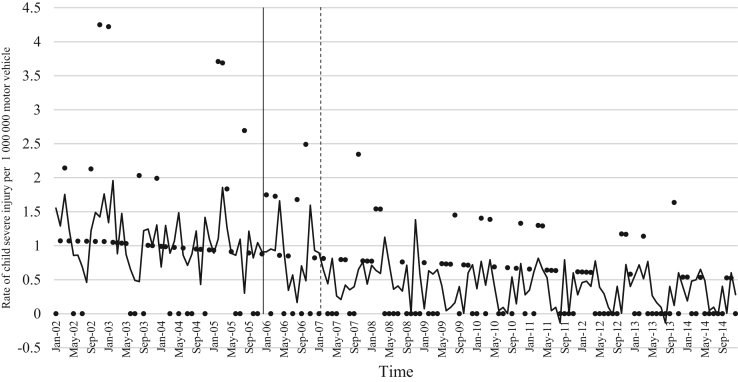
Rate of child severe injury per 1,000,000 motor vehicle in the Metropolitan Region 2002–2014. Data points represent monthly rates of child occupant severe injuries aged 0–3 between 2002 and 2014. The curved lines represent fitted values for seasonally adjusted models. The full vertical line represents the month of Chile׳s CRL implementation and the broken vertical line the introduction of Chile׳s National Decree.

**Fig. 6:**
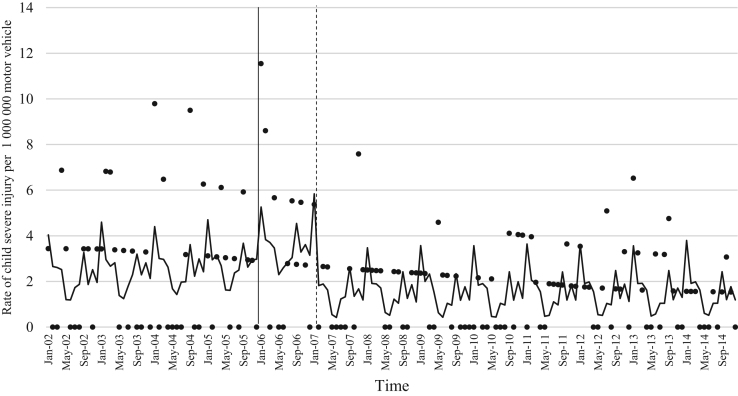
Rate of child severe injury per 1,000,000 motor vehicle in northern regions 2002–2014. Data points represent monthly rates of child occupant severe injuries aged 0–3 between 2002 and 2014. The curved lines represent fitted values for seasonally adjusted models. The full vertical line represents the month of Chile׳s CRL implementation and the broken vertical line the introduction of Chile׳s National Decree.

**Fig. 7:**
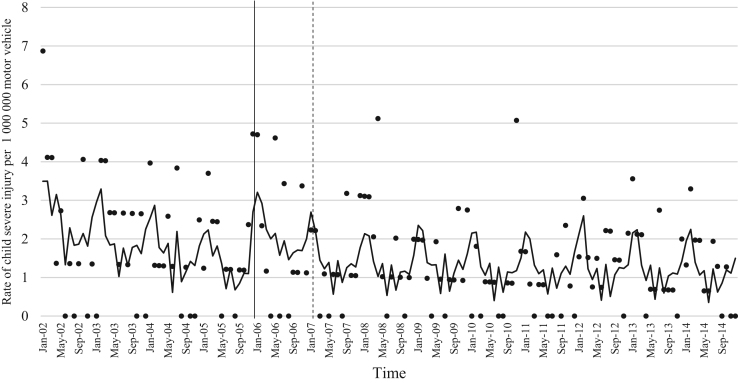
Rate of child severe injury per 1,000,000 motor vehicle in central regions 2002–2014. Data points represent monthly rates of child occupant severe injuries aged 0–3 between 2002 and 2014. The curved lines represent fitted values for seasonally adjusted models. The full vertical line represents the month of Chile׳s CRL implementation and the broken vertical line the introduction of Chile׳s National Decree.

**Fig. 8:**
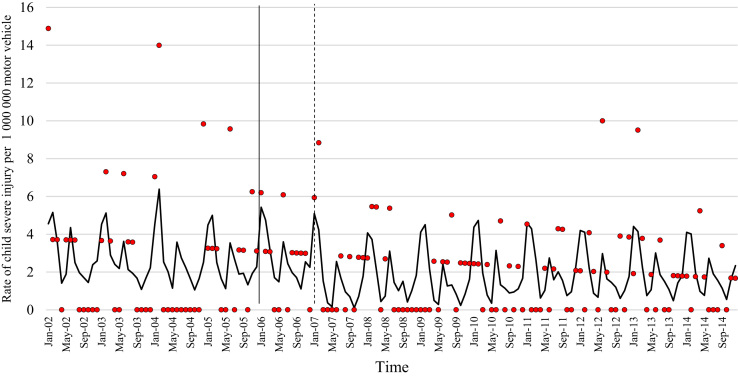
Rate of child severe injury per 1,000,000 motor vehicle in southern regions 2002–2014. Data points represent monthly rates of child occupant severe injuries aged 0–3 between 2002 and 2014. The curved lines represent fitted values for seasonally adjusted models. The full vertical line represents the month of Chile׳s CRL implementation and the broken vertical line the introduction of Chile׳s National Decree.

The data comprised of police data on the influence of child restraint legislation on reducing traffic child injuries and or fatalities for the period 2002–2014. Table 1 shows the descriptive statistics of the study variables. Table 2, Table 3, Table 4, Table 5, Table 6, Table 7, Table 8, Table 9, Table 10, Table 11, Table 12, Table 13, Table 14, Table 15 include ARIMA models considering linear, quadratic and logarithmic equations when assessing the impact of child restraint legislation for children per vehicle fleet. Table 16, Table 17, Table 18, Table 19, Table 20, Table 21, Table 22, Table 23, Table 24, Table 25 include Poisson models considering linear, quadratic and logarithmic equations when assessing the impact of child restraint legislation for children per vehicle fleet. Table 26, Table 27 describe the results with children per children population. Table 28, Table 29 describe the results in which traffic crashes and injuries are introduced as controls when assessing the ARIMA models. All tables contain a series of dummy variables representing each month of year were used to control for seasonality patterns. Seasonality is a condition that may affect traffic fatalities and injuries variation since in certain months of the year it can be observed more collisions due to higher traffic volume than in other months. Fig. 1, Fig. 2, Fig. 3, Fig. 4, Fig. 5, Fig. 6, Fig. 7, Fig. 8 depict fitted and raw data of child injuries and fatalities for Chile including several regions.

**Table 1:** Descriptive statistics of study variables, Chile January 2002–December 2014 (*N* = 156).

Variables	Mean	SD	Min	Max
*Dependent variables*				
Traffic fatalities of children (aged 0–3) per 1,000,000 motor vehicles in Chile	0.34	0.37	0	1.70
Traffic fatalities of children (aged 0–3) per 1,000,000 motor vehicles in the Metropolitan Region	0.13	0.33	0	1.79
Traffic fatalities of children (aged 0–3) per 1,000,000 motor vehicles in the Northern regions	0.80	1.46	0	6.79
Traffic fatalities of children (aged 0–3) per 1,000,000 motor vehicles in the Central regions	0.31	0.68	0	4.95
Traffic fatalities of children (aged 0–3) per 1,000,000 motor vehicles in the Southern regions	0.53	1.36	0	8.51
Traffic fatalities of children (aged 4–7) per 1,000,000 motor vehicles in Chile	0.30	0.33	0	1.80
Traffic severe injuries of children (aged 0–3) per 1,000,000 motor vehicles in Chile	1.32	0.91	0	4.95
Traffic severe injuries of children (aged 0–3) per 1,000,000 motor vehicles in the Metropolitan Region	0.63	0.81	0	4.24
Traffic severe injuries of children (aged 0–3) per 1,000,000 motor vehicles in the Northern regions	2.00	2.28	0	11.54
Traffic severe injuries of children (aged 0–3) per 1,000,000 motor vehicles in the Central regions	2.60	2.12	0	15.03
Traffic severe injuries of children (aged 0–3) per 1,000,000 motor vehicles in the Southern regions	2.16	2.73	0	14.88
Traffic severe injuries of children (aged 4–7) per 1,000,000 motor vehicles in Chile	2.16	1.27	0	7.11

**Table 2:** Effect of CRL and National Decree on traffic fatalities of children (aged 0–3) per 1,000,000 motor vehicles nationally.

	**Linear**			**Logarithmic**			**Quadratic**		
	***β***	**95% CI**		***β***	**95% CI**		***β***	**95% CI**	
Law (December 2005)	−0.30	−0.65	0.04	−0.21	−0.52	0.09	−0.28	−0.63	0.07
National Decree (February 2007)	0.12	−0.17	0.43	0.15	−0.15	0.46	0.09	−0.23	0.42
Time x National Decree	−0.01	−0.01	0.00	−0.00	−0.01	0.00	−0.00	−0.02	0.01
Time (trend)	0.00	−0.00	0.01	0.04	−0.11	0.420	0.01	−0.00	0.01
Time (quadratic)	–			–			−0.00	−0.00	0.00
Constant	0.37***	0.02	0.72	0.34	−0.23	0.92	0.36***	0.01	0.71
AR1 parameter	0.18***	0.02	0.33	0.18**	0.03	0.33	0.17**	0.02	0.33
**Goodness-of-fit**									
AIC	125.92			127.58			127.55		
BIC	185.27			190.06			190.03		
**Test for autocorrelation**									
Q (lag 1)	0.07; *p* = 0.78			0.06; *p* = 0.79			0.06; *p* = 0.79		
Q (lag 12)	21.34; *p* = 0.05			22.00; *p* = 0.04			21.83; *p* = 0.03		

All models contain 11 dummy variables to control for monthly variations and traffic fatalities of children population (aged 4–7) per vehicle fleet.

CI Confidence Interval; AR Autoregressive; AIC Akaike information criterion; BIC Bayesian information criterion; Q Portmanteau for white noise.

*** 1% significance level.

** 5% significance level.

**Table 3:** Effect of CRL and National Decree on traffic fatalities of children (aged 0–3) per 1,000,000 motor vehicles in the Metropolitan Region.

	**Linear**			**Logarithmic**			**Quadratic**		
	***β***	**95% CI**		***β***	**95% CI**		***β***	**95% CI**	
Law (December 2005)	−0.03	−0.32	0.25	0.09	−0.15	0.34	−0.07	−0.35	0.20
National Decree (February 2007)	−0.08	−0.30	0.13	−0.05	−0.26	0.15	−0.01	−0.23	0.21
Time x National Decree	−0.01	−0.02	0.00	−0.00	−0.01	0.00	−0.01	−0.03	0.00
Time (trend)	0.00	−0.00	0.02	0.08	−0.10	0.27	0.00	−0.00	0.01
Time (quadratic)	–			–			0.00	−0.00	0.00
Constant	−0.20	−0.67	0.26	0.26	−0.96	0.42	−0.18	−0.64	0.26
AR1 parameter	0.01	−0.19	0.21	0.03	−0.17	0.23	−0.00	−0.19	0.19
**Goodness-of-fit**									
AIC	114.19			116.35			113.99		
BIC	173.43			175.60			176.35		
**Test for autocorrelation**									
Q (lag 1)	0.00; *p* = 0.98			0.00; *p* = 0.96			0.00; *p* = 1.00		
Q (lag 12)	22.09; *p* = 0.03			20.93; *p* = 0.05			21.44; *p* = 0.04		

All models contain 11 dummy variables to control for monthly variations and traffic fatalities of children population (aged 4–7) per vehicle fleet. *** 1% significance level; ** 5% significance level; CI Confidence Interval; AR Autoregressive; AIC Akaike information criterion; BIC Bayesian information criterion; Q Portmanteau for white noise.

**Table 4:** Effect of CRL and National Decree on traffic fatalities of children (aged 0–3) per 1,000,000 motor vehicles in the Northern regions.

	**Linear**			**Logarithmic**			**Quadratic**		
	***β***	**95% CI**		***β***	**95% CI**		***β***	**95% CI**	
Law (December 2005)	0.14	−1.56	1.85	−0.13	−0.15	0.34	0.22	−1.50	1.94
National Decree (February 2007)	0.87	−0.31	2.06	0.79	−0.34	1.96	0.73	−0.54	2.01
Time x National Decree	0.01	−0.02	0.05	−0.00	−0.01	0.00	0.02	−0.04	0.10
Time (trend)	−0.02	−0.05	0.02	−0.18	−0.84	0.47	0.00	−0.00	0.01
Time (quadratic)	–			–			−0.00	−0.00	0.00
Constant	1.44**	0.24	2.65	1.61	−0.56	3.79	1.41**	0.19	2.63
**Goodness-of-fit**									
AIC	603.54			604.15			605.09		
BIC	659.78			660.38			664.44		
**Test for autocorrelation**									
Q (lag 1)	0.30; *p* = 0.58			0.35; *p* = 0.54			0.23; *p* = 0.62		
Q (lag 12)	13.64; *p* = 0.32			13.68; *p* = 0.32			13.70; *p* = 0.31		

All models contain 11 dummy variables to control for monthly variations and traffic fatalities of children population (aged 4–7) per vehicle fleet.

*** 1% significance level.

CI Confidence Interval; AR Autoregressive; AIC Akaike information criterion; BIC Bayesian information criterion; Q Portmanteau for white noise.

** 5% significance level.

**Table 5:** Effect of CRL and National Decree on traffic fatalities of children (aged 0–3) per 1,000,000 motor vehicles in the Central regions.

	**Linear**			**Logarithmic**			**Quadratic**		
	***β***	**95% CI**		***β***	**95% CI**		***β***	**95% CI**	
Law (December 2005)	−0.25	−1.05	0.55	−0.29	−0.91	0.33	−0.24	−1.07	0.59
National Decree (February 2007)	0.18	−0.48	0.85	0.17	−0.47	0.82	0.16	−0.61	0.93
Time x National Decree	−0.00	−0.02	0.01	−0.00	−0.01	0.01	0.00	−0.05	0.05
Time (trend)	0.00	−0.01	0.02	0.04	−0.18	0.27	0.00	−0.02	0.02
Time (quadratic)	–			–			−0.00	−0.00	0.00
Constant	0.69**	0.08	1.30	0.57	−0.21	1.35	0.68**	0.19	2.63
AR1 parameter	0.02	−0.14	0.19	0.02	−0.14	0.19	0.02	−0.14	0.20
AR2 parameter	0.16**	0.01	0.31	0.16**	0.02	0.30	0.16**	0.01	0.31
**Goodness-of-fit**									
AIC	365.22			365.09			367.17		
BIC	427.69			427.57			432.78		
**Test for autocorrelation**									
Q (lag 1)	0.00; *p* = 0.98			0.00; *p* = 0.98			0.00; *p* = 0.98		
Q (lag 12)	15.43; *p* = 0.21			15.64; *p* = 0.20			15.31; *p* = 0.22		

All models contain 11 dummy variables to control for monthly variations and traffic fatalities of children population (aged 4–7) per vehicle fleet.

*** 1% significance level.

CI Confidence Interval; AR Autoregressive; AIC Akaike information criterion; BIC Bayesian information criterion; Q Portmanteau for white noise.

** 5% significance level.

**Table 6:** Effect of CRL and National Decree on traffic fatalities of children (aged 0–3) per 1,000,000 motor vehicles in the Southern regions.

	**Linear**			**Logarithmic**			**Quadratic**		
	***β***	**95% CI**		***β***	**95% CI**		***β***	**95% CI**	
Law (December 2005)	−1.78***	−3.05	−0.52	−1.25**	−2.43	−0.07	−1.63**	−2.90	−0.36
National Decree (February 2007)	−0.35	−1.75	1.04	−0.20	−1.60	1.20	−0.64	−2.37	1.09
Time x National Decree	−0.03	−0.06	0.00	−0.00	−0.01	0.01	0.00	−0.08	0.09
Time (trend)	0.03**	0.00	0.07	0.52	−0.01	1.05	0.04**	0.01	0.08
Time (quadratic)	–			–			−0.00	−0.00	0.00
Constant	−0.09	−1.73	1.54	−0.77	−2.91	1.37	0.68**	0.19	2.63
AR1 parameter	−0.02	−0.20	0.15	−0.02	−0.21	0.16	0.02	−0.14	0.20
**Goodness-of-fit**									
AIC	593.04			594.15			592.73		
BIC	652.40			653.51			655.20		
**Test for autocorrelation**									
Q (lag 1)	0.00; *p* = 0.99			0.00; *p* = 0.99			0.00; *p* = 0.99		
Q (lag 12)	13.57; *p* = 0.32			13.64; *p* = 0.32			14.75; *p* = 0.25		

All models contain 11 dummy variables to control for monthly variations and traffic fatalities of children population (aged 4–7) per vehicle fleet.

CI Confidence Interval; AR Autoregressive; AIC Akaike information criterion; BIC Bayesian information criterion; Q Portmanteau for white noise.

*** 1% significance level.

** 5% significance level.

**Table 7:** Effect of CRL and National Decree on traffic severe injuries of children (aged 0–3) per 1,000,000 motor vehicles in Chile.

	**Linear**			**Logarithmic**			**Quadratic**		
	***β***	**95% CI**		***β***	**95% CI**		***β***	**95% CI**	
Law (December 2005)	0.23	−0.44	0.91	0.26	−0.26	0.80	0.22	−0.48	0.94
National Decree (February 2007)	−0.58**	−1.31	−0.02	−0.59**	−1.13	−0.04	−0.55	−1.18	0.06
Time x National Decree	0.00	−0.01	0.02	0.00	−0.00	0.00	0.00	−0.04	0.04
Time (trend)	−0.01	−0.02	0.00	−0.17	−0.35	0.01	−0.00	−0.02	0.01
Time (quadratic)	–			–			0.00	−0.00	0.00
Constant	1.58***	0.86	2.29	2.01***	1.19	2.83	1.59***	0.87	2.30
AR1 parameter	−0.04	−0.19	0.10	−0.03	−0.18	0.11	−0.04	−0.19	0.10
AR2 parameter	0.13	−0.04	0.32	0.13	−0.04	0.32	0.13	0.04	0.32
**Goodness-of-fit**									
AIC	407.76			407.77			409.73		
BIC	470.24			470.25			475.33		
**Test for autocorrelation**									
Q (lag 1)	0.01; *p* = 0.89			0.01; *p* = 0.89			0.01: *p* = 0.89		
Q (lag 12)	17.76; *p* = 0.12			17.76; *p* = 0.12			17.78; *p* = 0.12		

All models contain 11 dummy variables to control for monthly variations and traffic severe injuries of children population (aged 4–7) per vehicle fleet.

CI Confidence Interval; AR Autoregressive; AIC Akaike information criterion; BIC Bayesian information criterion; Q Portmanteau for white noise.

*** 1% significance level.

** 5% significance level.

**Table 8:** Effect of CRL and National Decree on traffic severe injuries of children (aged 0–3) per 1,000,000 motor vehicles in the Metropolitan Region.

	**Linear**			**Logarithmic**			**Quadratic**		
	***β***	**95% CI**		***β***	**95% CI**		***β***	**95% CI**	
Law (December 2005)	−0.09	−0.71	0.53	−0.19	−0.75	0.36	−0.06	−0.71	0.57
National Decree (February 2007)	−0.31	−0.90	0.28	−0.33	−0.92	0.25	−0.36	−1.02	0.30
Time x National Decree	0.00	−0.01	0.02	0.00	−0.00	0.00	0.00	−0.03	0.04
Time (trend)	−0.01	−0.01	0.01	0.09	−0.10	0.29	0.00	−0.02	0.02
Time (quadratic)	–			–			−0.00	−0.00	0.00
Constant	0.70**	0.11	1.29	0.39	−0.35	1.15	0.68**	0.08	1.28
AR1 parameter	0.01	−0.15	0.17	0.00	−0.16	0.17	0.00	−0.16	0.17
AR2 parameter	0.08	−0.07	0.23	0.08	−0.07	0.23	0.08	−0.07	0.23
**Goodness-of-fit**									
AIC	386.14			385.58			387.97		
BIC	448.50			447.94			453.45		
**Test for autocorrelation**									
Q (lag 1)	0.01; *p* = 0.91			0.01; *p* = 0.91			0.01; *p* = 0.91		
Q (lag 12)	14.65; *p* = 0.26			13.39; *p* = 0.34			14.90; *p* = 0.24		

All models contain 11 dummy variables to control for monthly variations and traffic severe injuries of children population (aged 4–7) per vehicle fleet. ***1% significance level.

CI Confidence Interval; AR Autoregressive; AIC Akaike information criterion; BIC Bayesian information criterion; Q Portmanteau for white noise.

** 5% significance level.

**Table 9:** Effect of CRL and National Decree on traffic severe injuries per 1,000,000 motor vehicles in the Northern regions.

	**Linear**			**Logarithmic**			**Quadratic**		
	***β***	**95% CI**		***β***	**95% CI**		***β***	**95% CI**	
Law (December 2005)	0.55	−0.79	1.90	0.59	−0.41	1.58	0.61	−0.81	2.05
National Decree (February 2007)	−1.98***	−3.20	−0.76	−1.96***	−3.12	−0.79	−2.10***	−3.50	−0.69
Time x National Decree	−0.01	−0.05	0.02	−0.00	−0.01	0.01	0.00	−0.03	0.04
Time (trend)	0.01	−0.02	0.05	0.34	−0.23	0.93	0.01	−0.11	0.11
Time (quadratic)	–			–			−0.00	−0.00	0.00
Constant	1.71	0.11	1.29	0.99	−1.29	3.28	1.66**	0.03	3.29
AR1 parameter	−0.11	−0.25	0.02	−0.11	−0.25	0.01	−0.11	−0.25	0.01
**Goodness-of-fit**									
AIC	749.25			748.45			751.07		
BIC	808.61			807.81			813.55		
**Test for autocorrelation**									
Q (lag 1)	0.04; *p* = 0.83			0.05; *p* = 0.82			0.04; *p* = 0.82		
Q (lag 12)	12.80; *p* = 0.38			12.74; *p* = 0.38			13.16; *p* = 0.35		

All models contain 11 dummy variables to control for monthly variations and traffic severe injuries of children population (aged 4–7) per vehicle fleet.

CI Confidence Interval; AR Autoregressive; AIC Akaike information criterion; BIC Bayesian information criterion; Q Portmanteau for white noise.

*** 1% significance level.

** 5% significance level.

**Table 10:** Effect of CRL and National Decree on traffic severe injuries of children (aged 0–3) per 1,000,000 motor vehicles in the Central regions.

	**Linear**			**Logarithmic**			**Quadratic**		
	***β***	**95% CI**		***β***	**95% CI**		***β***	**95% CI**	
Law (December 2005)	1.04	−0.12	2.22	0.86**	0.04	1.68	1.00	−0.19	2.20
National Decree (February 2007)	−0.33	−1.18	−0.52	−0.44	−1.22	0.34	−0.24	−1.17	−0.67
Time x National Decree	0.02	−0.00	0.05	0.00	−0.01	0.01	0.01	−0.05	0.08
Time (trend)	−0.02	−0.05	0.00	−0.61***	−0.99	0.23	−0.03	−0.06	0.00
Time (quadratic)	–			–			0.00	−0.00	0.00
Constant	2.51***	1.47	3.55	3.69***	2.30	5.09	2.54***	1.50	3.58
AR1 parameter	−0.03	−0.21	0.14	−0.03	−0.21	0.13	−0.03	−0.21	0.14
AR2 parameter	0.16**	0.01	0.31	0.16**	0.01	0.32	0.16**	0.01	0.32
**Goodness-of-fit**									
AIC	555.97			552.13			557.77		
BIC	618.45			614.61			623.37		
**Test for autocorrelation**									
Q (lag 1)	0.19; *p* = 0.65			0.18; *p* = 0.66			0.19: *p* = 0.65		
Q (lag 12)	17.07; *p* = 0.14			17.94; *p* = 0.11			17.33; *p* = 0.13		

All models contain 11 dummy variables to control for monthly variations and traffic severe injuries of children population (aged 4–7) per vehicle fleet.

CI Confidence Interval; AR Autoregressive; AIC Akaike information criterion; BIC Bayesian information criterion; Q Portmanteau for white noise.

*** 1% significance level.

** 5% significance level.

**Table 11:** Effect of CRL and National Decree on traffic severe injuries of children (aged 0–3) per 1,000,000 motor vehicles in the Southern regions.

	**Linear**			**Logarithmic**			**Quadratic**		
	***β***	**95% CI**		***β***	**95% CI**		***β***	**95% CI**	
Law (December 2005)	0.08	−2.45	2.22	0.69	−1.54	2.93	−0.07	−2.65	2.49
National Decree (February 2007)	−1.02	−3.35	1.31	−0.88	−3.16	1.38	−0.72	−3.18	1.74
Time x National Decree	0.02	−0.04	0.05	0.01	−0.01	0.03	−0.03	−0.15	0.09
Time (trend)	−0.00	−0.05	0.04	−0.62**	−1.21	−0.03	−0.01	−0.07	0.04
Time (quadratic)	–			–			0.00	−0.00	0.00
Constant	2.27***	0.16	4.39	4.09***	1.74	6.44	2.35**	0.24	4.46
AR1 parameter	0.01	−0.17	0.20	0.02	−0.16	0.20	0.02	−0.17	0.21
**Goodness-of-fit**									
AIC	806.04			803.70			807.37		
BIC	865.40			863.06			869.85		
**Test for autocorrelation**									
Q (lag 1)	0.00; *p* = 0.98			0.00; *p* = 0.98			0.00; *p* = 0.98		
Q (lag 12)	6.26; *p* = 0.90			5.93; *p* = 0.91			6.75; *p* = 0.87		

All models contain 11 dummy variables to control for monthly variations and traffic severe injuries of children population (aged 4–7) per vehicle fleet.

CI Confidence Interval; AR Autoregressive; AIC Akaike information criterion; BIC Bayesian information criterion; Q Portmanteau for white noise.

*** 1% significance level.

** 5% significance level.

**Table 12:** Effect of CRL and National Decree on traffic fatalities of children (aged 4–7) per 1,000,000 motor vehicles in Chile.

	**Linear**			**Logarithmic**			**Quadratic**		
	***β***	**95% CI**		***β***	**95% CI**		***β***	**95% CI**	
Law (December 2005)	0.31**	0.05	0.57	0.21**	0.00	0.41	0.31**	0.04	0.57
National Decree (February 2007)	−0.00	−0.18	0.17	−0.03	−0.21	0.14	0.00	−0.19	0.20
Time x National Decree	0.01**	0.00	0.01	0.00	−0.01	0.01	0.00	−0.00	0.01
Time (trend)	−0.01	−0.01	−0.00	−0.00	−0.00	0.00	−0.01**	−0.01	−0.00
Time (quadratic)	–			–			−0.00	−0.00	0.00
Constant	0.40***	0.13	0.67	0.59***	0.21	0.96	0.40***	0.13	0.67
AR1 parameter	−0.13	−0.28	0.02	−0.13^*^	−0.28	0.02	−0.13	−0.28	0.02
**Goodness-of-fit**									
AIC	102.96			102.61			104.93		
BIC	162.32			161.97			167.41		
**Test for autocorrelation**									
Q (lag 1)	0.01; *p* = 0.90			0.01; *p* = 0.89			0.01; *p* = 0.89		
Q (lag 12)	8.86; *p* = 0.71			9.03; *p* = 0.69			8.89; *p* = 0.71		

All models contain 11 dummy variables to control for monthly variations and traffic severe injuries of children population (aged 0–3) per vehicle fleet.

CI Confidence Interval; AR Autoregressive; AIC Akaike information criterion; BIC Bayesian information criterion; Q Portmanteau for white noise.

*** 1% significance level.

** 5% significance level.

**Table 13:** Effect of CRL and National Decree on traffic severe injuries of children (aged 4–7) per 1,000,000 motor vehicles in Chile.

	**Linear**			**Logarithmic**			**Quadratic**		
	***β***	**95% CI**		***β***	**95% CI**		***β***	**95% CI**	
Law (December 2005)	1.12***	0.42	1.81	0.59**	0.05	1.13	1.05***	0.31	1.80
National Decree (February 2007)	−0.30	−0.90	0.29	−0.47	−1.05	0.10	−0.17	−0.79	0.44
Time x National Decree	0.03***	0.01	0.05	−0.00	−0.00	0.00	0.02	−0.02	0.06
Time (trend)	−0.04***	−0.05	−0.02	−0.73***	−0.94	−0.53	−0.04***	−0.06	−0.02
Time (quadratic)	–			–			0.00	−0.00	0.00
Constant	4.12***	3.13	5.11	4.09***	1.74	6.44	4.16**	3.16	5.16
AR1 parameter	−0.13	−0.28	0.02	−0.16**	−0.31	−0.01	−0.13	−0.28	0.00
**Goodness-of-fit**									
AIC	480.69			477.31			481.65		
BIC	540.05			536.67			544.13		
**Test for autocorrelation**									
Q (lag 1)	0.03; *p* = 0.84			0.03; *p* = 0.85			0.03; *p* = 0.85		
Q (lag 12)	18.91; *p* = 0.09			20.19; *p* = 0.06			20.08; *p* = 0.06		

All models contain 11 dummy variables to control for monthly variations and traffic severe injuries of children population (aged 4–7) per vehicle fleet.

CI Confidence Interval; AR Autoregressive; AIC Akaike information criterion; BIC Bayesian information criterion; Q Portmanteau for white noise.

*** 1% significance level

** 5% significance level.

**Table 14:** Effect of CRL and National Decree on traffic fatalities of children (aged 4–7) per 1,000,000 motor vehicles in Chile.

	**Linear**			**Logarithmic**			**Quadratic**		
	***β***	**95% CI**		***β***	**95% CI**		***β***	**95% CI**	
Law (December 2005)	0.31**	0.05	0.57	0.21**	0.00	0.41	0.31**	0.04	0.57
National Decree (February 2007)	−0.00	−0.18	0.17	−0.03	−0.21	0.14	0.00	−0.19	0.20
Time x National Decree	0.01**	0.00	0.01	0.00	−0.01	0.01	0.00	−0.00	0.01
Time (trend)	−0.01	−0.01	−0.00	−0.00	−0.00	0.00	−0.01**	−0.01	−0.00
Time (quadratic)	–			–			−0.00	−0.00	0.00
Constant	0.40***	0.13	0.67	0.59***	0.21	0.96	0.40***	0.13	0.67
AR1 parameter	−0.13	−0.28	0.02	−0.13^*^	−0.28	0.02	−0.13	−0.28	0.02
**Goodness-of-fit**									
AIC	102.96			102.61			104.93		
BIC	162.32			161.97			167.41		
**Test for autocorrelation**									
Q (lag 1)	0.01; *p* = 0.90			0.01; *p* = 0.89			0.01; *p* = 0.89		
Q (lag 12)	8.86; *p* = 0.71			9.03; *p* = 0.69			8.89; *p* = 0.71		

All models contain 11 dummy variables to control for monthly variations and traffic severe injuries of children population (aged 0–3) per vehicle fleet.

CI Confidence Interval; AR Autoregressive; AIC Akaike information criterion; BIC Bayesian information criterion; Q Portmanteau for white noise.

*** 1% significance level.

** 5% significance level.

**Table 15:** Effect of CRL and National Decree on traffic severe injuries of children (aged 4–7) per 1,000,000 motor vehicles in Chile.

	**Linear**			**Logarithmic**			**Quadratic**		
	***β***	**95% CI**		***β***	**95% CI**		***β***	**95% CI**	
Law (December 2005)	1.12***	0.42	1.81	0.59**	0.05	1.13	1.05***	0.31	1.80
National Decree (February 2007)	−0.30	−0.90	0.29	−0.47	−1.05	0.10	−0.17	−0.79	0.44
Time x National Decree	0.03***	0.01	0.05	−0.00	−0.00	0.00	0.02	−0.02	0.06
Time (trend)	−0.04***	−0.05	−0.02	−0.73***	−0.94	−0.53	−0.04***	−0.06	−0.02
Time (quadratic)	–			–			0.00	−0.00	0.00
Constant	4.12***	3.13	5.11	4.09***	1.74	6.44	4.16**	3.16	5.16
AR1 parameter	−0.13	−0.28	0.02	−0.16**	−0.31	−0.01	−0.13	−0.28	0.00
**Goodness-of-fit**									
AIC	480.69			477.31			481.65		
BIC	540.05			536.67			544.13		
**Test for autocorrelation**									
Q (lag 1)	0.03; *p* = 0.84			0.03; *p* = 0.85			0.03; *p* = 0.85		
Q (lag 12)	18.91; *p* = 0.09			20.19; *p* = 0.06			20.08; *p* = 0.06		

All models contain 11 dummy variables to control for monthly variations and traffic severe injuries of children population (aged 4–7) per vehicle fleet.

CI Confidence Interval; AR Autoregressive; AIC Akaike information criterion; BIC Bayesian information criterion; Q Portmanteau for white noise.

*** 1% significance level.

** 5% significance level.

**Table 16:** Effect of CRL and National Decree on traffic fatalities of children (aged 0–3) per 1,000,000 motor vehicles nationally (Poisson).

	**Linear**			**Logarithmic**			**Quadratic**		
	***β***	**95%**	**CI**	***β***	**95%**	**CI**	***β***	**95%**	**CI**
Law (December 2005)	−0.66	−1.57	0.23	−0.52	−1.23	0.19	−0.63	−1.54	0.28
National Decree (February 2007)	0.34	−0.28	0.97	0.39	−0.21	0.99	0.09	−0.23	0.42
Time x National Decree	−0.01	−0.03	0.00	−0.00	−0.01	−0.00	−0.00	−0.04	0.03
Time (trend)	0.00	−0.01	0.03	−0.14	−0.21	0.49	0.01	−0.01	0.03
Time (quadratic)	–			–			−0.00	−0.00	0.00
Constant	−14.89**	−15.58	−14.20	15.07***	−16.17	13.97	0.36***	0.01	0.71
Lag of dependent variable	0.11***	0.01	0.22	0.11***	0.01	0.22	0.17**	0.02	0.33
**Goodness-of-fit**									
AIC	451.70			451.92			453.36		
BIC	507.83			508.05			512.60		

All models contain 11 dummy variables to control for monthly variations and traffic fatalities of children population (aged 4–7) per vehicle fleet.

CI Confidence Interval; AIC Akaike information criterion; BIC Bayesian information criterion.

*** 1% significance level.

** 5% significance level.

**Table 17:** Effect of CRL and National Decree on traffic fatalities of children (aged 0–3) per 1,000,000 motor vehicles in the Metropolitan Region (Poisson).

	**Linear**			**Logarithmic**			**Quadratic**		
	***β***	**95%**	**CI**	***β***	**95%**	**CI**	***β***	**95%**	**CI**
Law (December 2005)	−1.38	−4.04	1.27	−1.43	−4.57	1.70	−1.55	−4.27	1.16
National Decree (February 2007)	−0.71	−1.98	0.55	−0.72	−1.96	0.51	−0.29	−1.74	1.15
Time x National Decree	−0.12	−0.22	−0.02	−0.13	−0.31	0.05	−0.18	−0.33	−0.03
Time (trend)	0.11	0.01	0.20	−0.30	−4.49	3.88	0.08	−0.01	0.17
Time (quadratic)	–			–			0.00	−0.00	0.00
Constant	−21.13***	−25.54	−16.72	15.07***	−16.17	13.97	−20.72***	−24.87	−16.57
Lag of dependent variable	0.39	−0.34	1.12	0.38	−0.35	1.12	0.35	−0.43	1.33
**Goodness-of-fit**									
AIC	160.12			162.11			160.72		
BIC	214.90			219.94			218.54		

All models contain 11 dummy variables to control for monthly variations and traffic fatalities of children population (aged 4–7) per vehicle fleet.

**5% significance level; CI Confidence Interval; AR Autoregressive; AIC Akaike information criterion; BIC Bayesian information criterion; Q Portmanteau for white noise.

*** 1% significance level.

**Table 18:** Effect of CRL and National Decree on traffic fatalities of children (aged 0–3) per 1,000,000 motor vehicles in the Northern regions (Poisson).

	**Linear**			**Logarithmic**			**Quadratic**		
	***β***	**95%**	**CI**	***β***	**95%**	**CI**	***β***	**95%**	**CI**
Law (December 2005)	0.49	−1.59	2.58	0.95	−1.64	3.55	0.72	−1.39	2.84
National Decree (February 2007)	1.02	0.01	2.04	1.19	0.08	2.30	0.77	−0.31	1.86
Time x National Decree	0.01	−0.02	0.06	0.04	−0.04	0.13	0.06	−0.02	0.15
Time (trend)	−0.02	−0.06	0.01	0.43	−0.56	1.42	−0.01	−0.06	0.03
Time (quadratic)	–			–			0.00	−0.00	0.00
Constant	−13.51***	−14.62	−12.39	14.02***	−15.41	−12.62	−13.57***	−14.65	−12.48
**Goodness-of-fit**									
AIC	247.13			248.65			247.94		
BIC	298.97			303.55			302.84		

All models contain 11 dummy variables to control for monthly variations and traffic fatalities of children population (aged 4–7) per vehicle fleet.

**5% significance level; CI Confidence Interval; AIC Akaike information criterion; BIC Bayesian information criterion.

*** 1% significance level.

**Table 19:** Effect of CRL and National Decree on traffic fatalities of children (aged 0–3) per 1,000,000 motor vehicles in the Central regions (Poisson).

	**Linear**			**Logarithmic**			**Quadratic**		
	***β***	**95%**	**CI**	***β***	**95%**	**CI**	***β***	**95%**	**CI**
Law (December 2005)	−0.55	−2.33	1.23	−0.68	−2.16	0.79	−0.30	−2.14	1.52
National Decree (February 2007)	0.89	−0.46	2.25	0.85	−0.48	2.20	0.47	−1.02	1.98
Time x National Decree	−0.01	−0.04	0.03	−0.01	−0.04	0.00	0.06	−0.04	0.17
Time (trend)	−0.00	−0.04	0.03	−0.04	−0.78	0.70	0.02	−0.03	0.07
Time (quadratic)	–			–			−0.00	−0.00	0.00
Constant	−13.95**	−15.11	−12.78	13.96***	−16.33	−11.06	−14.22***	−15.57	−12.86
Lag 1 of dependent variable	−0.01	−0.38	0.35	−0.01	−0.38	0.35	0.17**	0.02	0.33
Lag 2 of dependent variable	0.29	−0.10	0.69	0.28	−0.11	0.35			
**Goodness-of-fit**									
AIC	240.97			241.03			240.68		
BIC	298.67			298.73			301.42		

All models contain 11 dummy variables to control for monthly variations and traffic fatalities of children population (aged 4–7) per vehicle fleet.

CI Confidence Interval; AIC Akaike information criterion; BIC Bayesian information criterion.

*** 1% significance level.

** 5% significance level.

**Table 20:** Effect of CRL and National Decree on traffic fatalities of children (aged 0–3) per 1,000,000 motor vehicles in the Southern regions (Poisson).

	**Linear**			**Logarithmic**			**Quadratic**		
	***β***	**95%**	**CI**	***β***	**95%**	**CI**	***β***	**95%**	**CI**
Law (December 2005)	−2.75**	−5.18	−0.33	−2.34**	−4.58	−0.11	−1.84	−4.34	0.66
National Decree (February 2007)	−0.33	−2.49	1.82	−0.24	−2.40	1.90	−3.06	−6.79	0.66
Time x National Decree	−0.03	−0.07	0.00	−0.01	−0.04	0.00	0.23	0.01	0.44
Time (trend)	0.04**	0.00	0.08	0.93	−0.36	2.22	0.13**	0.04	0.21
Time (quadratic)	–			–			−0.00**	−0.00	−0.00
Constant	−15.24**	−16.73	−13.75	16.99***	−21.36	−12.63	−16.24***	−18.23	−14.24
Lag 1 of dependent variable	−0.10	−0.85	0.64	−0.12	−0.86	0.61	−0.21	−0.92	0.48
**Goodness-of-fit**									
AIC	184.38			184.22			177.30		
BIC	239.16			239.00			235.13		

All models contain 11 dummy variables to control for monthly variations and traffic fatalities of children population (aged 4–7) per vehicle fleet.

CI Confidence Interval; AIC Akaike information criterion; BIC Bayesian information criterion.

*** 1% significance level.

** 5% significance level.

**Table 21:** Effect of CRL and National Decree on traffic injuries of children (aged 0–3) per 1,000,000 motor vehicles nationally (Poisson).

	**Linear**			**Logarithmic**			**Quadratic**		
	***β***	**95%**	**CI**	***β***	**95%**	**CI**	***β***	**95%**	**CI**
Law (December 2005)	0.01	−0.40	0.43	0.01	−0.30	0.33	0.01	−0.40	0.43
National Decree (February 2007)	−0.45**	−0.78	−0.12	−0.45**	−0.77	−0.13	−0.45**	−0.82	−0.08
Time x National Decree	−0.00	−0.01	0.01	−0.00	−0.01	0.01	−0.00	−0.04	0.03
Time (trend)	0.00	−0.01	0.02	0.02	−0.13	0.19	0.01	−0.01	0.03
Time (quadratic)	–			–			−0.00	−0.00	0.00
Constant	−13.89**	−13.94	−12.83	15.07***	−16.17	13.97	13.38**	−13.95	−12.81
Lag of dependent variable	0.00	−0.03	0.03	0.11***	0.01	0.22	0.17**	0.02	0.33
**Goodness-of-fit**									
AIC	733.75			733.69			735.75		
BIC	789.88			789.81			794.99		

All models contain 11 dummy variables to control for monthly variations and traffic fatalities of children population (aged 4–7) per vehicle fleet.

CI Confidence Interval; AR Autoregressive; AIC Akaike information criterion; BIC Bayesian information criterion; Q Portmanteau for white noise.

*** 1% significance level.

** 5% significance level.

**Table 22:** Effect of CRL and National Decree on traffic injuries of children (aged 0–3) per 1,000,000 motor vehicles in the Metropolitan Region (Poisson).

	**Linear**			**Logarithmic**			**Quadratic**		
	***β***	**95%**	**CI**	***β***	**95%**	**CI**	***β***	**95%**	**CI**
Law (December 2005)	−0.14	−0.98	0.68	−0.06	−1.06	0.94	−0.02	−0.91	0.86
National Decree (February 2007)	−0.26	−0.88	0.34	−0.24	−0.88	0.39	−0.43	−1.15	0.28
Time x National Decree	−0.00	−0.02	0.02	0.05	−0.03	0.04	0.27	−0.03	0.08
Time (trend)	0.00	−0.02	0.01	0.14	−0.46	0.75	0.08	−0.01	0.17
Time (quadratic)	–			–			−0.00	−0.00	0.00
Constant	−13.8***	−14.65	−12.94	14.02***	−15.20	−12.84	−13.87***	−14.74	−12.99
Lag 2 of dependent variable	0.01	−0.14	0.17	0.01	−0.15	0.17	0.01	−0.14	0.17
**Goodness-of-fit**									
AIC	360.34			362.24			361.55		
BIC	415.00			419.94			419.26		

All models contain 11 dummy variables to control for monthly variations and traffic fatalities of children population (aged 4–7) per vehicle fleet.

**5% significance level; CI Confidence Interval; AR Autoregressive; AIC Akaike information criterion; BIC Bayesian information criterion; Q Portmanteau for white noise.

*** 1% significance level.

**Table 23:** Effect of CRL and National Decree on traffic injuries of children (aged 0–3) per 1,000,000 motor vehicles in the Northern regions (Poisson).

	**Linear**			**Logarithmic**			**Quadratic**		
	***β***	**95%**	**CI**	***β***	**95%**	**CI**	***β***	**95%**	**CI**
Law (December 2005)	0.27	−0.50	1.06	0.27	−0.56	1.29	0.37	−0.41	1.16
National Decree (February 2007)	−0.81**	−1.39	−0.23	−0.81**	−1.36	−0.21	−1.01**	−1.75	−0.26
Time x National Decree	−0.00	−0.02	0.02	0.00	−0.04	0.05	0.27	−0.03	0.08
Time (trend)	0.00	−0.01	0.02	0.05	−0.46	0.75	0.01	−0.01	0.03
Time (quadratic)	–			–			−0.00	−0.00	0.00
Constant	−13.19***	−14.08	−12.30	13.32***	−14.86	−11.77	−13.29***	−14.20	−12.38
Lag 2 of dependent variable	−0.13	−0.32	0.05	−0.13	−0.32	0.05	−0.14	−0.32	0.04
**Goodness-of-fit**									
AIC	377.79			377.76			379.00		
BIC	432.46			432.43			436.70		

All models contain 11 dummy variables to control for monthly variations and traffic fatalities of children population (aged 4–7) per vehicle fleet.

CI Confidence Interval; AR Autoregressive; AIC Akaike information criterion; BIC Bayesian information criterion; Q Portmanteau for white noise.

*** 1% significance level.

** 5% significance level.

**Table 24:** Effect of CRL and National Decree on traffic injuries of children (aged 0–3) per 1,000,000 motor vehicles in the Central regions (Poisson).

	**Linear**			**Logarithmic**			**Quadratic**		
	***β***	**95%**	**CI**	***β***	**95%**	**CI**	***β***	**95%**	**CI**
Law (December 2005)	0.27	−0.34	0.90	0.17	−0.33	0.67	0.27	−0.34	0.92
National Decree (February 2007)	−0.18	−0.68	0.32	−0.21	−0.71	0.29	−0.17	−0.72	0.37
Time x National Decree	−0.00	−0.01	0.01	−0.00	−0.00	0.00	0.00	−0.04	0.04
Time (trend)	−0.00	−0.02	0.00	−0.08	−0.35	0.17	−0.01	−0.02	0.01
Time (quadratic)	–			–			−0.00	−0.00	0.00
Constant	−13.15***	−13.81	−12.50	13.05***	−14.06	−12.05	−13.15***	−13.82	−12.48
Lag 1 of dependent variable	−0.00	−0.08	0.07	−0.00	−0.08	0.07	−0.00	−0.08	0.07
Lag 2 of dependent variable	0.05	−0.03	0.14	0.05	−0.03	0.14	0.05	−0.03	0.14
**Goodness-of-fit**									
AIC	487.63			487.82			489.63		
BIC	545.33			545.52			550.37		

All models contain 11 dummy variables to control for monthly variations and traffic fatalities of children population (aged 4–7) per vehicle fleet.

**5% significance level; CI Confidence Interval; AIC Akaike information criterion; BIC Bayesian information criterion.

*** 1% significance level.

**Table 25:** Effect of CRL and National Decree on traffic injuries of children (aged 0–3) per 1,000,000 motor vehicles in the Southern regions (Poisson).

	**Linear**			**Logarithmic**			**Quadratic**		
	***β***	**95%**	**CI**	***β***	**95%**	**CI**	***β***	**95%**	**CI**
Law (December 2005)	−0.19	−0.99	0.59	−0.00	−0.60	0.60	−0.24	−1.07	0.58
National Decree (February 2007)	−0.62**	−1.19	−0.04	−0.56**	−1.12	−0.00	−0.55	−1.23	0.12
Time x National Decree	−0.01	−0.03	0.01	−0.00	−0.00	0.00	−0.02	−0.08	0.04
Time (trend)	0.01	−0.00	0.03	0.12	−0.19	0.44	0.00	−0.01	0.03
Time (quadratic)	–			–			0.00	−0.00	0.00
Constant	−13.33***	−14.14	−12.52	−13.42**	−14.47	−12.37	−13.31***	−14.13	−12.50
Lag 1 of dependent variable	−0.04	−0.22	0.12	−0.04	−0.22	0.12	−0.05	−0.22	0.12
**Goodness-of-fit**									
AIC	374.62			375.02			376.49		
BIC	429.40			429.81			434.32		

All models contain 11 dummy variables to control for monthly variations and traffic fatalities of children population (aged 4–7) per vehicle fleet.

CI Confidence Interval; AIC Akaike information criterion; BIC Bayesian information criterion.

*** 1% significance level.

** 5% significance level.

**Table 26:** Effect of CRL and National Decree on traffic fatalities of children (aged 0–3) per 1,000,000 child population in Chile, the Metropolitan Region, Northern regions, Central regions and Southern regions.

	**National**			**Metropolitan Region**			**Northern regions**			**Central regions**			**Southern regions**		
	***β***	**95% CI**		***β***	**95% CI**		***β***	**95% CI**		***β***	**95% CI**		***β***	**95% CI**	
Law (December 2005)	−0.43	−1.13	0.25	−0.10	−0.96	0.74	0.30	−6.03	6.64	−0.62	−3.79	2.54	−3.34	−7.11	0.42
National Decree (February 2007)	0.22	−0.33	0.78	−0.19	−0.83	0.44	2.51	−1.71	6.75	0.67	−1.87	3.21	−0.66	−4.81	3.48
Time x National Decree	−0.01	−0.01	0.00	−0.01	−0.04	0.00	0.01	−0.13	0.18	−0.00	−0.09	0.09	−0.05	−0.16	0.06
Time (trend)^a^	0.00	−0.00	0.01	0.00	−0.00	0.04	−0.03	−0.18	0.12	0.00	−0.08	0.08	0.06	−0.04	0.17
Constant	0.51	−0.24	1.27	−0.56	−1.95	0.82	3.21	−1.60	8.02	2.07	−1.45	5.60	−0.04	−5.09	5.00
AR1 parameter	0.18***	0.02	0.33	−0.27	−0.56	0.00	–			0.18	−2.21	2.58	−0.20	−0.79	0.37
AR2 parameter	–			–			–			0.08	0.83	0.99	–		
**Test for autocorrelation**															
Q (lag 1)	0.02; *p* = 0.88			0.05; *p* = 0.81			0.00; *p* = 0.97			0.00; *p* = 0.96			0.00; *p* = 0.97		
Q (lag 12)	19.86; *p* = 0.06			13.85; *p* = 0.31			14.06; *p* = 0.29			14.38; *p* = 0.27			10.41; *p* = 0.57		

All models contain 11 dummy variables to control for monthly variations and traffic fatalities of children population (aged 4–7) per children population.

** 5% significance level; CI Confidence Interval; AR Autoregressive; Q Portmanteau for white noise.

*** 1% significance level.

**Table 27:** Effect of CRL and National Decree on traffic severe injuries of children (aged 0–3) per 1,000,000 population in Chile, the Metropolitan Region, Northern regions, Central regions and Southern regions.

	**National**			**Metropolitan Region**			**Northern regions**			**Central regions**			**Southern regions**		
	***β***	**95% CI**		***β***	**95% CI**		***β***	**95% CI**		***β***	**95% CI**		***β***	**95% CI**	
Law (December 2005)	0.45	−1.89	2.80	−0.11	−1.66	1.43	2.16	−2.59	6.92	0.80	−2.71	4.32	1.53	−11.69	8.63
National Decree (February 2007)	−1.99**	−3.68	−0.30	−0.70	−2.17	0.74	−5.39*	−11.44	0.66	−0.79	−2.89	1.30	−3.94	−11.87	3.98
Time x National Decree	0.00	−0.01	0.02	0.00	−0.03	0.04	−0.00	−0.01	0.01	0.00	−0.01	0.01	−0.04	−0.34	0.26
Time (trend)	0.04	−0.02	0.00	0.00	−0.03	0.03	0.04	−0.08	0.17	−0.61***	−0.99	0.23	0.09	−0.19	0.39
Constant	3.69***	1.29	60.8	1.01	−1.94	3.97	3.33	−4.09	10.76	3.52**	0.08	6.96	1.85	−9.34	13.05
AR1 parameter	0.32**	0.01	0.62	−0.27	−0.93	0.38	−0.17	−1.10	0.75	−0.30**	−0.61	0.00	0.18	−0.25	0.62
AR2 parameter	–			0.03	−0.34	0.41	−0.12	−0.38	0.12				–		
**Test for autocorrelation**															
Q (lag 1)	0.00; *p* = 0.98			0.00; *p* = 0.92			0.00; *p* = 0.99			0.04; *p* = 0.83			0.02; *p* = 0.89		
Q (lag 12)	10.75; *p* = 0.55			11.12; *p* = 0.51			12.74; *p* = 0.38			17.37; *p* = 0.11			10.35; *p* = 0.58		

All models contain 11 dummy variables to control for monthly variations and traffic injuries of children population (aged 4–7) per children population.

CI Confidence Interval; AR Autoregressive; Q Portmanteau for white noise.

*** 1% significance level.

** 5% significance level.

**Table 28:** Effect of CRL and National Decree on traffic fatalities of children (aged 0–3) per 1,000,000 vehicles controlling for All injuries (Model 1) and All crashes (Model 2).

	**Model 1 With all injuries**			**Model 2 With all crashes**		
	***β***	**95% CI**		***β***	**95% CI**	
Law (December 2005)	−0.26	−0.65	0.12	−0.30	−0.70	0.09
National Decree (February 2007)	0.09	−0.21	0.40	0.10	−0.25	0.46
Time x National Decree	−0.00	−0.01	0.01	−0.01	−0.01	0.00
Time (trend)	0.00	−0.00	0.01	0.00	−0.00	0.01
Total injuries	0.03	−0.03	0.10			
Total crashes				0.00	−0.00	0.01
Constant	0.25	−1.93	1.42	0.30	−0.95	1.57
AR1 parameter	0.19**	0.03	0.34	0.18**	0.01	0.35
**Test for autocorrelation**						
Q (lag 1)	0.07; *p* = 0.78			0.01; *p* = 0.89		
Q (lag 12)	20.49; *p* = 0.06			19.40; *p* = 0.08		

All models contain 11 dummy variables to control for monthly variations total injury rates (all population all ages) per population in the population model. *** 1% significance level.

CI Confidence Interval; AR Autoregressive; Q Portmanteau for white noise.

** 5% significance level.

**Table 29:** Effect of CRL and National Decree on traffic severe of children (aged 0–3) per 1,000,000 vehicles controlling for All injuries (Model 1) and All crashes (Model 2).

	**Model 1 Total injuries**			**Model 2 Total crashes**		
	***β***	**95% CI**		***β***	**95% CI**	
Law (December 2005)	0.09	−0.84	1.17	0.40	−0.27	1.08
National Decree (February 2007)	−0.81**	−1.52	−0.10	−0.66**	−1.23	−0.09
Time x National Decree	−0.00	−0.02	0.02	0.01	−0.01	0.03
Time (trend)	−0.00	−0.00	0.01	−0.01	−0.03	0.00
Total injuries	0.02	−0.13	0.19			
Total crashes				0.00	−0.01	0.01
Constant	3.31	−0.38	7.01	1.66	−0.43	3.77
AR1 parameter	0.30**	0.00	0.61	−0.03	−0.18	0.11
AR2 parameter				0.13	−0.05	0.32
**Test for autocorrelation**						
Q (lag 1)	0.00; *p* = 0.98			0.00; *p* = 0.93		
Q (lag 12)	15.84; *p* = 0.19			18.56; *p* = 0.09		

All models contain 11 dummy variables to control for monthly variations total injury rates (all population all ages) per population in the population model. *** 1% significance level.

CI Confidence Interval; AR Autoregressive; Q Portmanteau for white noise.

** 5% significance level.

## 2 Experimental design, materials and methods

Since each time series has a unique structure, ARIMA models are developed using a three-stage iterative process: (1) identification, (2) estimation and (3) diagnostic [2]. Identification involves examining both autocorrelation and partial autocorrelation matrices to establish, first, whether the series was stochastic or deterministic, and, second, autoregressive and moving average parameters [3], [4].

We explain the ARIMA model selection processes for two different dependent variables which correspond to Chile and Chile׳s Metropolitan Region. We provide the explanation of the selection process for traffic fatalities at the national level (Chile), and traffic injuries for the Metropolitan Region.

### 2.1 Traffic fatalities at the national level

To identifying the order of differencing in the ARIMA model for traffic fatalities at the national level, we proceed by testing first the stationarity of the time series for the variable traffic fatalities in Chile. Two tests can be applied to identify the realization of a stationary process Dickey–Fuller [4], [5] and Phillips–Perron [6]. Results of these tests displayed in Table 30 suggest that the time-series of this variable have a stationary process, and therefore the series do not require to be differentiated. In order to confirm the latter, we proceed to analyze the autocorrelation of residuals for 40 lags for this variable.

**Table 30:** Dickey–Fuller and Phillips–Perron tests to identify stationarity in traffic fatalities at the national level.

**Test**	**Test statistic**	***p*-value**
Dickey–Fuller	−117.964	0.000
Phillips–Perron	−9.564	0.000

As we can observe the distribution of the autocorrelations of the residuals has both negative and positive results confirming a stationary process in the series.

**Fig. 9:**
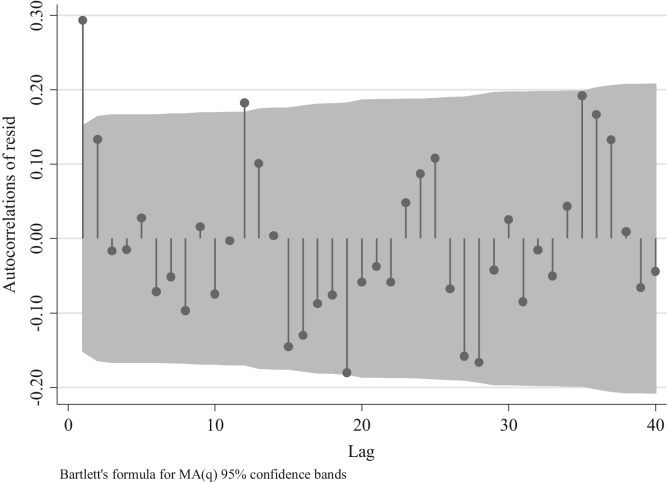
Distribution of Autocorrelations of residuals in traffic fatalities at the national level.

To identify the AR and/or MA terms for the ARIMAs model for traffic fatalities at the national level we inspect the distribution of the residuals depicted in Fig. 9. This figure suggests the presence of an AR model rather than a MA one, since the distribution of the residuals after the first two lags is different from 0 [3]. Nevertheless, we can compare four models one with the absence of AR and MA terms (ARIMA (0,0,0), two with different AR terms ((1,0,0) and (2,0,0)), and one with an MA term (0,0,1) to confirm what we observed in Fig. 9. For this we analyze the partial autocorrelation of residuals in traffic fatalities at the national level. To identify an MA term one should observe a decaying process with negative values, whereas for the AR terms one should observe spikes in different lags which will determine the number of terms.

**Fig. 10:**
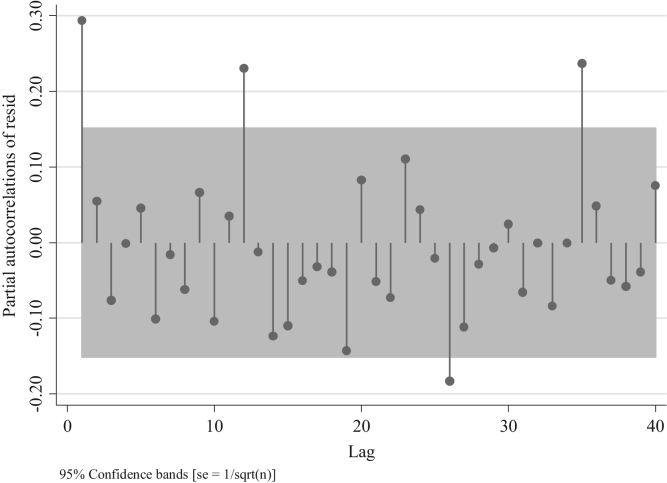
Distribution of Partial autocorrelations of residuals in traffic fatalities at the national level for an ARIMA (0,0,0).

**Fig. 11:**
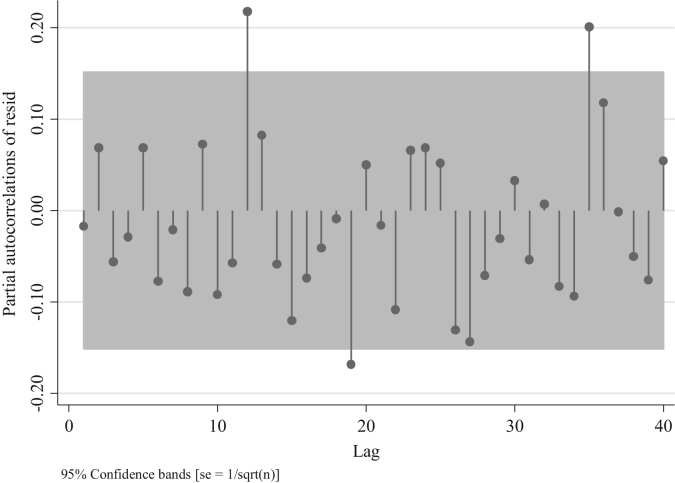
Distribution of Partial autocorrelations of residuals in traffic fatalities at the national level for an ARIMA (1,0,0).

**Fig. 12:**
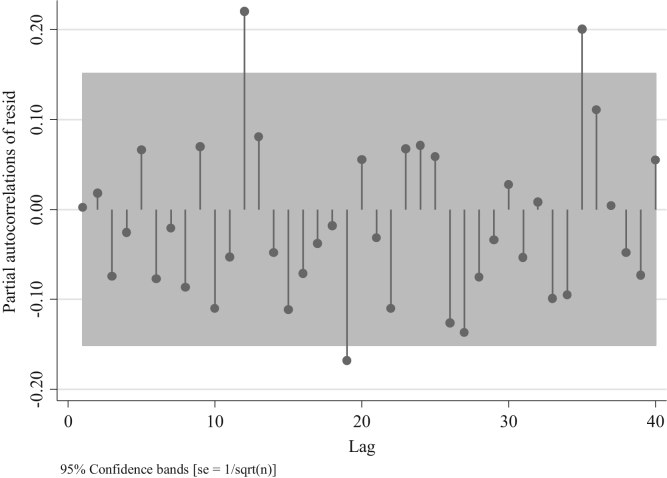
Distribution of Partial autocorrelations of residuals in traffic fatalities at the national level for an ARIMA (2,0,0).

**Fig. 13:**
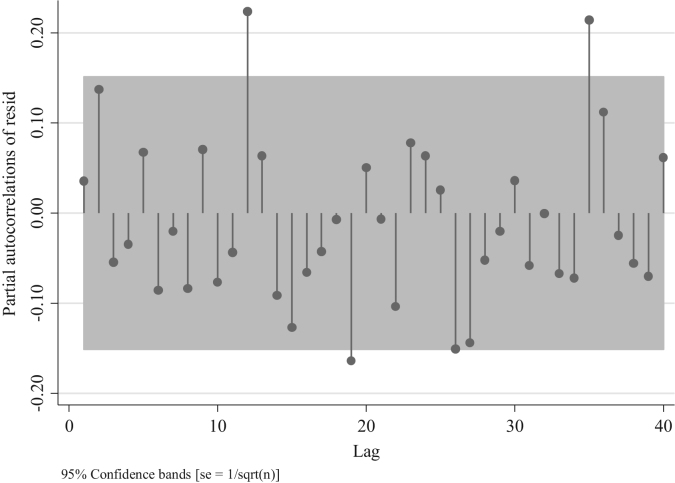
Distribution of Partial autocorrelations of residuals in traffic fatalities at the national level for an ARIMA (0,0,1).

We observe in Fig. 10 the presence of a positive partial autocorrelation suggesting an AR model rather than an MA. This is confirmed in Figs. 11 and 12, in which partial autocorrelation of residuals are not present in the first lag. In Fig. 13 we also observe the same pattern when we model an ARIMA (0,0,1).

This leads us to test the presence of white noise in each of these four models. In order to determine the white noise we provide the value for the Portmanetu (Q) test [7] in Table 31. A value lower than 0.05 identifies autocorrelation and therefore the confidence intervals of the estimators of interests are biased. Table 31 confirms the presence of a model with an AR term since the p values for the ARIMA (1,0,0) and ARIMA (2,0,0) models are higher than 0.05. In order to determine which model fits the data better, we proceed to compare the AIC and BIC values for each model. We observe in Table 32 that the ARIMA (1,0,0) model fits the data better, and therefore we can proceed to analyze traffic fatality variation with this model.

**Table 31:** Portmanteau test for white noise for models ARIMA (0,0,0); ARIMA (1,0,0); ARIMA (2,0,0); and ARIMA (0,0,1).

	**ARIMA (0,0,0)**	**ARIMA (1,0,0)**	**ARIMA (2,0,0)**	**ARIMA (0,0,1)**
Portmanteau (Q)	85.76, *p* = 0.00	52.87, *p* = 0.08	54.88, *p* = 0.05	55.92, *p* = 0.04

**Table 32:** AIC and BIC values for ARIMA (1,0,0) and ARIMA (2,0,0) models.

	**ARIMA (1,0,0)**	**ARIMA (2,0,0)**
AIC	136.90	138.39
BIC	146.27	150.89

### 2.2 Traffic injuries for the Metropolitan Region

To identify whether the distribution of traffic injuries over time in the Metropolitan Region follows or not a stationary process we apply two tests, Dickey–Fuller and Phillips–Perron. In Table 33, we display the results for each test. Results of both tests suggest that the time-series of traffic injuries in the Metropolitan Region have a stationary process, and therefore the series do not require to be differentiated ( Tables 34 and 35).

**Table 33:** Dickey–Fuller and Phillips–Perron tests to identify stationarity in traffic injuries in the Metropolitan Region.

**Test**	**Test statistic**	***p*-value**
Dickey–Fuller	−156.660	0.000
Phillips–Perron	−10.933	0.000

**Table 34:** Portmanteau test for white noise for models ARIMA (0,0,0); ARIMA (1,0,0); ARIMA (2,0,0); and ARIMA (0,0,1).

	**ARIMA (0,0,0)**	**ARIMA (1,0,0)**	**ARIMA (2,0,0)**	**ARIMA (0,0,1)**
Portmanteau (Q)	82.23, *p* = 0.00	56.32, *p* = 0.04	32.30, *p* = 0.80	64.34, *p* = 0.00

**Table 35:** AIC and BIC values for ARIMA (2,0,0) and ARIMA (3,0,0) models.

	**ARIMA (1,0,0)**	**ARIMA (2,0,0)**
AIC	408.13	398.77
BIC	417.50	411.26

**Fig. 14:**
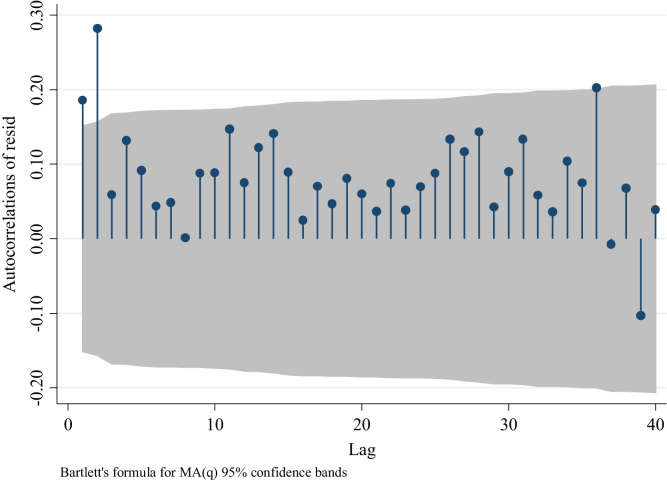
Distribution of Autocorrelations of residuals in traffic injuries in the Metropolitan Region for an ARIMA (0,0,0).

We observe in Fig. 14, the autocorrelation of the residuals is positive in the first two lags, and then the autocorrelations of the residuals are not decaying consequently, but rather show a fluctuating patter. This suggest that the series may not need to be differentiated.

**Fig. 15:**
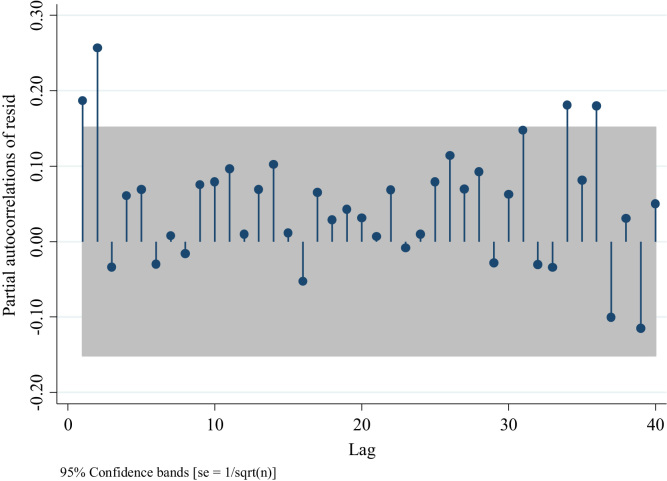
Distribution of Partial autocorrelations of residuals in traffic injuries in the Metropolitan Region for an ARIMA (0,0,0).

**Fig. 16:**
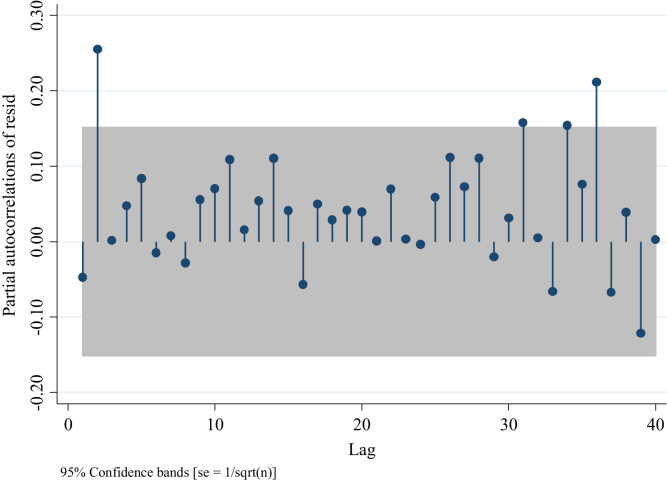
Distribution of Partial autocorrelations of residuals in traffic injuries in the Metropolitan Region for an ARIMA (1,0,0).

Similarly, to the series of traffic fatalities at the national level (Fig. 9), Fig. 14 suggests also the presence of an AR model rather than an MA one, since the distribution of the residuals after the first two lags is different from 0 [3]. Nevertheless, we can compare four models one with the absence of AR and MA terms ARIMA (0,0,0), two with different AR terms ((1,0,0) and (2,0,0)), and one with an MA term (0,0,1) to confirm what we observed in Fig. 14. For this we analyze the partial autocorrelation of residuals in traffic injuries for the Metropolitan Region. To identify an MA term, one should observe a decaying process with negative values, whereas for the AR terms one should observe spikes in different lags which will determine the number of terms ( Figs. 15,16 and 18).

**Fig. 17:**
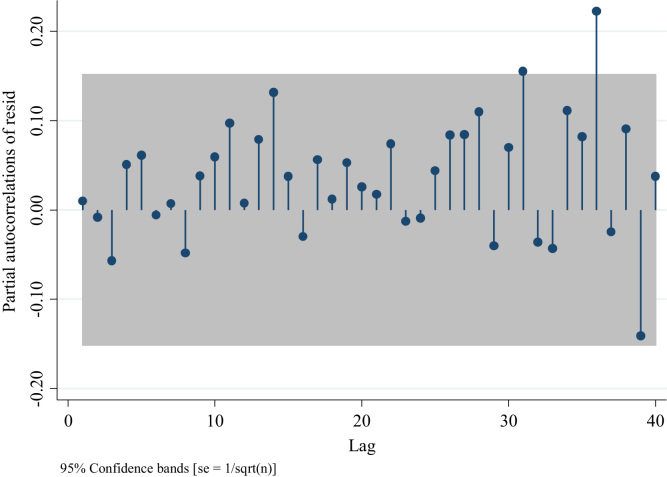
Distribution of Partial autocorrelations of residuals in traffic injuries for the Metropolitan Region for an ARIMA (2,0,0).

Out of these four figures only Fig. 17 displays partial autocorrelations of residuals not significant for the first 12 lags. This confirms the presence of an AR model. To determine the white noise for these models in the following table we provide the value for the Portmanetu (Q) tests. In which a value lower than 0.05 identifies autocorrelation and therefore the confidence intervals of the estimators of interests are biased.

**Fig. 18:**
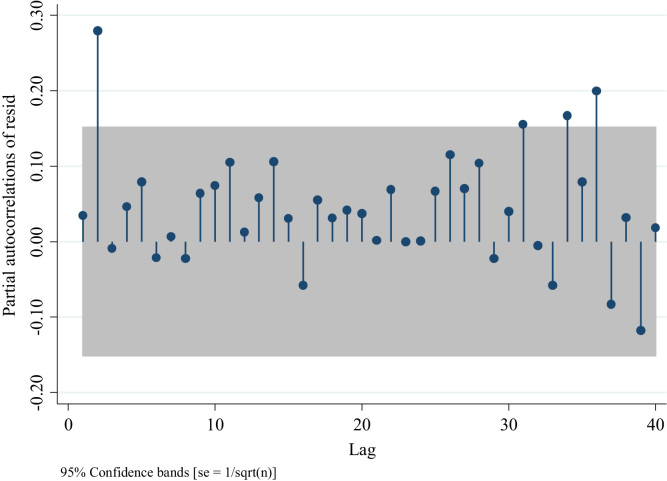
Distribution of Partial autocorrelations of residuals in traffic injuries for the Metropolitan Region ARIMA (0,0,1).

According to these values only the ARIMA (2,0,0,) fits the data better for these series. Since the partial test associated to the presence of white noise is not significant at *p* < 0.05. To confirm this result, we report the AIC and BIC values of the ARIMA (1,0,0) and ARIMA (2,0,0) models, since these two had the highest *p*-values.

Following Burnham and Anderson [8] we observe that the ARIMA (2,0,0) model fits the data better than the ARIMA (1,0,0) model since its both AIC and BIC values are lower than the ones corresponding to the ARIMA (3,0,0). In sum the model ARIMA (2,0,0) is chosen to compare with an alternative ARIMA model with an MA part identified.

In sum the ARIMA (2,0,0) model is the most appropriate to assess traffic injury variation in the Metropolitan Region.
