# Supplementary material for: Dataset and figures on time-series analysis of child restraint policy impact in Chile
Source: Data Brief. 2018 Nov 22;21:2290–315. doi: 10.1016/j.dib.2018.11.079 (PMC6279945; doi:10.1016/j.dib.2018.11.079)
Supplement: Supplementary file 1 — Supplementary material [file mmc1.pdf]

The authors of the manuscript ‘Dataset and figures on time-series analysis of child restraint policy impact in Chile’ declare having no conflicts of interests with the content of the abovementioned article.

José Ignacio Nazif-Muñoz [a], Arijit Nandi [a], and Mónica Ruiz-Casares [b]

Affiliations:

[a] Institute for Health and Social Policy and Department of Epidemiology, Biostatistics, and Occupational Health, McGill University, Montreal, Quebec, Canada

[b] Department of Psychiatry, McGill University, Montreal, Quebec, Canada

Contact email: jose.nazifmunoz@mail.mcgill.ca
